# Assessment of the Causal Effects of Obstructive Sleep Apnea on Atrial Fibrillation: A Mendelian Randomization Study

**DOI:** 10.3389/fcvm.2022.843681

**Published:** 2022-02-11

**Authors:** Yalan Li, Yiming Leng, Haibo Tang, Peizhi Deng, Jie Wang, Hong Yuan, Rujia Miao, Ping Mu

**Affiliations:** ^1^Health Management Center, The Third Xiangya Hospital, Central South University, Changsha, China; ^2^Clinical Research Center, The Third Xiangya Hospital, Central South University, Changsha, China; ^3^Department of Metabolic and Bariatric Surgery, The Third Xiangya Hospital, Central South University, Changsha, China; ^4^Department of Biochemistry and Molecular Biology, Shenyang Medical College, Shenyang, China

**Keywords:** obstructive sleep apnea, atrial fibrillation, Mendelian randomization, causal inference, instrumental variable (IV)

## Abstract

**Background:**

Obstructive sleep apnea (OSA) and atrial fibrillation (AF) are epidemiologically correlated, but the causal relationship between them remains elusive. We aimed to explore the causal relationships between OSA and AF.

**Method:**

Using both the Finnish biobank and publicly available genome-wide association study data (GWAS), we conducted a two-sample Mendelian randomization (MR) analysis to estimate the causal effect of OSA on AF, both in the primary analysis and replicated analysis. The inverse variance weighted MR was selected as the main method. To further test the independent causal effect of OSA on AF, we also performed multivariable MR (MVMR), adjusting for body mass index (BMI), hypertension, and coronary artery disease (CAD), respectively.

**Results:**

In the primary analysis, OSA was significantly associated with the increased risk of AF (OR 1.21, 95% CI 1.11–1.32) and the replicated analysis showed consistent results (OR 1.17, 95% CI 1.05–1.30). Besides, there was no heterogeneity and horizontal pleiotropy observed both in the primary and replicated analysis. Further multivariable MR suggested that the causal relationships between OSA and AF exist independently of BMI and CAD. The MVMR result after the adjustment for hypertension is similar in magnitude and direction to the univariable MR. But it did not support a causal relationship between OSA and AF.

**Conclusion:**

Our study found that genetically driven OSA causally promotes AF. This causal relationship sheds new light on taking effective measures to prevent and treat OSA to reduce the risk of AF.

## Introduction

Atrial fibrillation (AF) is the most common type of arrhythmia that causes hemodynamic disorders, thrombotic strokes, heart failure, and increased mortality (1), with an estimated global prevalence of 34 million (2). Prevention of AF is important to improve the societal and personal costs related to the disease. AF is influenced by a number of non-genetic risk factors. In addition to traditional risk factors such as smoking, alcohol, and other lifestyle factors, as well as obesity, diabetes, and hypertension, a growing number of studies are focusing on the impacts of obstructive sleep apnea (OSA) on atrial fibrillation (3–6).

Notably, OSA is characterized by recurrent episodes of airway collapse during sleep, the combined prevalence of OSA in patients with AF has been estimated at 21 to 74% (6, 7). A few large longitudinal cohort studies recruiting a non-aged population reported that those with OSA, also inevitably featured with obesity, had a higher risk of incident AF than those without OSA after a follow-up of several years (8–10). Given that approximately 3–49% of the general population is estimated to be OSA (7), targeting OSA through airway pressure treatment or weight reduction has the potential to lower the incidence of AF if the observed associations between OSA and AF are causal. However, the previous studies are limited in assessing the comorbid confounder of overweight/obesity, a risk factor commonly shared by both OSA and AF (11), so that it is unknown whether OSA and AF have a direct causal relationship, or simply represent comorbidities that cluster in obese subjects. Furthermore, despite guidelines suggesting that OSA treatment might lower AF reoccurrence, the evidence is mainly based on small-sample observational studies or non-randomized clinical trials (12), which are prone to systematic biases and can not support a causal association.

Based on the deficiency of the former studies' design, we considered Mendelian randomization (MR). MR can be used to resolve causal assumptions. It is a genetic instrumental variable analysis that can evaluate the potential relationship between exposure and outcome (13). As MR uses instrumental variable analysis to model the randomization process that underlies causal inference in randomized controlled trials (RCTs), this design is less susceptible to confounding and reverse causality bias (14). In the current study, we aimed to perform two-sample MR analyses to test the hypothesis that there is a potential causality between OSA and AF.

## Methods

### Study Design Overview

**Figure 1:**
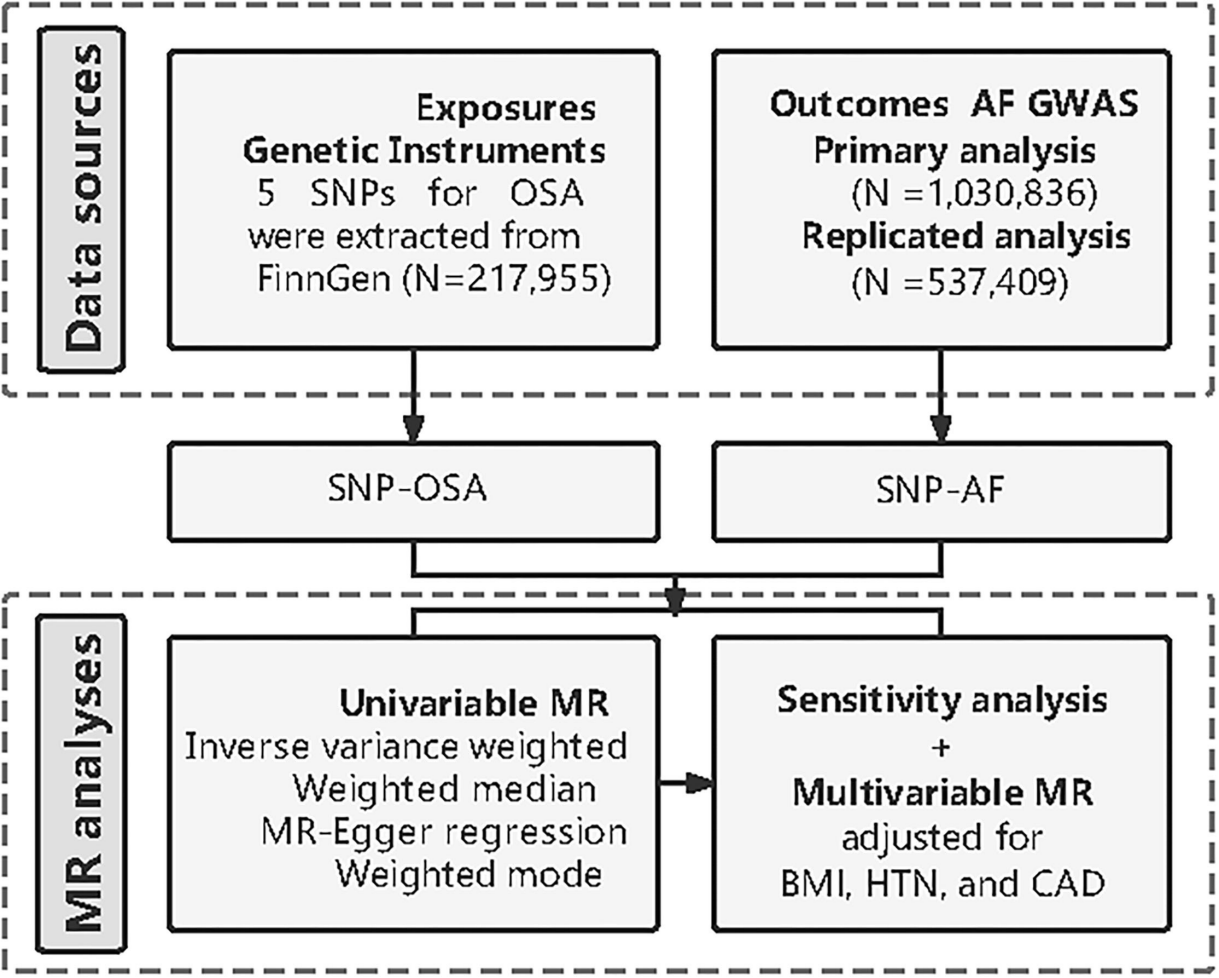
Diagram of Mendelian randomization (MR) framework in this study. SNP, single nucleotide polymorphisms; OSA, obstructive sleep apnea; AF, atrial fibrillation; GWAS, genome-wide association study; MR, mendelian randomization; BMI, body mass index; HTN, hypertension; CAD, coronary artery disease.

The overview of this study design is presented in Figure 1. Firstly, we extracted five available genetic instrumental variables from the FinnGEN consortium for OSA. Secondly, we obtained the summary data of AF from the genome-wide association study (GWAS). Then, we conducted univariable two-sample MR and several sensitivity analyses to estimate the causal effect. Finally, we carried out a multivariable MR (MVMR) analysis respectively adjusting for body mass index (BMI), hypertension, and coronary artery disease (CAD). In addition to the primary analysis, we constructed another AF dataset from a large meta-analysis to replicate the MR analysis to test our hypothesis.

### Data Source for Exposure

Our main exposure genetically determined OSA as an instrumental variable (IV). We extracted exposure data from the FINNGen consortium (https://www.finngen.fi/en), a large study aiming to collect and analyze genome and health data from 500,000 Finnish biobank participants. We identified 5 single nucleotide polymorphisms (SNP) robustly associated with OSA in the FINNGen genome-wide association study that included 217,955 participants (15), nearly all European ancestry (Supplementary Figure S1), with a statistically significant threshold [*P* < 5 × 10^−8^; linkage disequilibrium (LD); r^2^ < 0.001, LD distance > 10,000 kb]. F statistic {calculated as F = [(*n*-*k*-1)/k]/[*R*^2^/(1-*R*^2^)], the explained variance for exposure (*R*^2^), sample size (*n*), and number of SNPs (*k*)} indicates the strength of the relationship between SNP and OSA. In general, F-statistic >10 indicates a significant association between selected instrumental variables and OSA (16).

Furthermore, we obtained summary data on BMI, hypertension, and CAD from GWAS. The summary-level data for BMI were extracted from a meta-analysis of GWAS including 681,275 individuals of European ancestry (17). Data for hypertension was also obtained from the FinnGen consortium (22,154 cases and 74,345 controls) (https://www.finngen.fi/fi), nearly all of European ancestry. And the summary data on CAD we used was from a large meta-analysis of GWAS which includes 141,217 individuals of European ancestry (18).

### Data Source for the Outcome

We constructed two sets of outcome data for two MR analyses to test our hypothesis. For the primary analysis, the summary data for AF were derived from a GWAS meta-analysis involving nearly 1,030,836 European participants, including 60,620 cases and 970,216 controls (19). For the replication analysis, the summary data for AF were extracted from another GWAS meta-analysis, which investigated 537,409 participants of 84.2% European ancestry in up to 55,114 cases and 482,295 controls (20). To our knowledge, there was no sample overlap between the exposure and outcome GWASs.

### Statistical Analysis

#### Two-Sample Mendelian Randomization Analysis

As shown in Supplementary Figure S1, the conventional MR model was used to assess the causal effect of OSA on AF. The ideal instrumental variable must satisfy the following several requirements (Supplementary Figure S1): (i) the instrumental variables have to be closely related to OSA; (ii) there is no association between the instrumental variables and confounders of OSA and AF; (iii) the effect of instrumental variables on AF must be mediated solely by the OSA in the study. In the two-sample MR analysis, we used four different methods [inverse-variance weighted (IVW), weighted median, weight mode, and MR-Egger], to evaluate the causal effects between OSA and AF. And we used the MR-Pleiotropy Residual Sum and Outlier (MR-PRESSO) to address the problem of heterogeneity and pleiotropy effect. The results were shown as odds ratios (OR) and 95% CIs. The inverse-variance weighted method was used as the main analysis in our study. It is the most precise method for estimating causal effects (21). The weighted median estimator pools the median impact of SNPs with more than 50% of the weight coming from valid SNPs (22). The MR-Egger regression was used primarily to account for horizontal pleiotropy. Besides, we used the MR-PRESSO method to identify potential horizontal pleiotropy outliers in the analysis and adjust if necessary (23).

#### Sensitivity Univariable MR Analyses

To further explain the potential pleiotropy between exposure and outcome, a series of sensitivity analyses were conducted. Cochran's Q test was used to quantify the heterogeneity of instrumental variables, with P_h_ < 0.05 indicating heterogeneity (24). The Egger intercept in the MR Egger regression analysis was used as an indicator of horizontal pleiotropy (23). Furthermore, we conducted the leave-one-out analysis to remove a single SNP from the analysis in sequence. Both the primary and replicated analysis was conducted in the sensitivity analysis described above.

#### Multivariable Two-Sample MR Analysis

Previous observational studies have shown that BMI (25), hypertension (26), and CAD (27) were important risk factors for the development of atrial fibrillation, therefore, we further respectively performed an MVMR analysis to estimate the direct causal effect of OSA on the risk of AF. Both the primary and replicated analysis was conducted the MVMR described above.

Mendelian randomized (MR) analysis was performed in R (version 4.0.3; The R Foundation for Statistical Computing, Vienna, Austria, https://www.R-project.org/) with R packages “TwoSampleMR”, and “MVMR”. A two-tailed *p*-value < 0.05 was considered statistically significant.

#### Power Calculations

To evaluate the power of our study, a non-centrality parameter-based approach was used, which is a publicly available mRnd web tool (http://cnsgenomics.com/shiny/mRnd/) (28). For binary outcomes (AF), after we inputted the required parameters in mRnd (α = 0.05, R^2^ = 0.083 in this study), the minimum detectable OR was roughly estimated. The results of the power calculations are shown in Supplementary Table S2.

## Results

### The Character of SNP and Participants for Analysis

The characteristics of the populations included in the FINNGen and GWAS data of outcomes are shown in Table 1. We selected 5 SNP as instrumental variables for OSA. The F-statistic value >100 suggests that these selected SNPs are significantly associated with OSA in the MR analysis. The details of the available SNPs in the MR analysis are presented in Supplementary Table S1.

**Table 1:** Characteristics of obstructive sleep apnea and atrial fibrillation datasets.

**Exposure**	**Consortium**	**SNP*/F-*stat**	**Cases/Controls**	**Sample size**	**Female (%)**	**Population**
Obstructive sleep apnea	FINNGen	5/525	16,761/201,194	217,955	56.5%	European
**Outcome**	**Data source**	**Studies**	**Cases/Controls**	**Sample size**	**Female (%)**	**Population**
Primary analysis						
Atrial fibrillation	Meta-analysis	6	60,620/970,216	1,030,836	52.3%	European
Replicated analysis						
Atrial fibrillation	Meta-analysis	44	55,114/482,295	537,409	NA	84.2% European

*F-statistic, The smallest F-statistic of SNP in our study was 525. SNP, Single-nucleotide polymorphism*.

### Causal Effect of OSA on AF

#### Univariable Two-Sample MR Analysis Results

The overall results of the univariable MR are shown in Table 2. For the primary analysis, we observed a significant causal effect of OSA on AF (*P* < 0.05 in the four MR methods, including MR-Egger, IVW, Weighted median, and Weighted mode). OSA was associated with an increased risk of AF (OR 1.21, 95% CI 1.11–1.32). For the replicated analysis, there was still clear evidence to support a causal effect between OSA and AF (*P* < 0.05 in the IVW, Weighted median, and Weighted mode). The OR and 95%CI of OSA were 1.17 (1.05–1.30) for incident AF risk.

**Table 2:** Univariable and multivariable two-sample Mendelian randomization estimations showing the effect of obstructive sleep apnea on the risk of atrial fibrillation.

**Outcomes**	**Methods**	**Odds ratio (95% CI)**	***P*** **-value**	**Q-statistics**	***P***_**h**_	**Egger intercept**	***P***_***intercept***_
AF	MR-Egger	1.38 (1.14–1.66)	4.44E−02	3.03	3.86e−01	−0.014 (−0.033–0.005)	2.36e−01
Primary	Inverse-variance weighted	1.21 (1.11–1.32)	2.41E−05	5.23	2.64e−01		
	Weighted median	1.28 (1.15–1.43)	1.06E−05				
	Weighted mode	1.29 (1.14–1.47)	1.76E−02				
	MVMR (BMI adjusted)	1.10 (1.04–1.17)	9.81E−04				
	MVMR (HTN adjusted)	1.16 (0.91–1.48)	2.45E−01				
	MVMR (CAD adjusted)	1.17 (1.02–1.34)	2.62E−02				
AF	MR-Egger	1.17 (0.85–1.60)	4.02E−01	6.50	8.95e−02	0.00005 (−0.031–0.031)	9.98e−01
Replicated	Inverse-variance weighted	1.17 (1.05–1.30)	3.52E−03	6.50	1.64e−01		
	Weighted median	1.21 (1.08–1.35)	9.54E−04				
	Weighted mode	1.22 (1.06–1.39)	4.49E−02				
	MVMR (BMI adjusted)	1.10 (1.03–1.18)	4.70E−03				
	MVMR (HTN adjusted)	1.17 (0.92–1.49)	2.15E−01				
	MVMR (CAD adjusted)	1.18 (1.02–1.37)	2.92E−02				

*AF, atrial fibrillation; MR, mendelian randomization; MVMR, multivariable mendelian randomization; BMI, body mass index; HTN, hypertension; CAD, coronary artery disease; CI, cofidence interval*.

#### Sensitivity Analysis Validation

**Figure 2:**
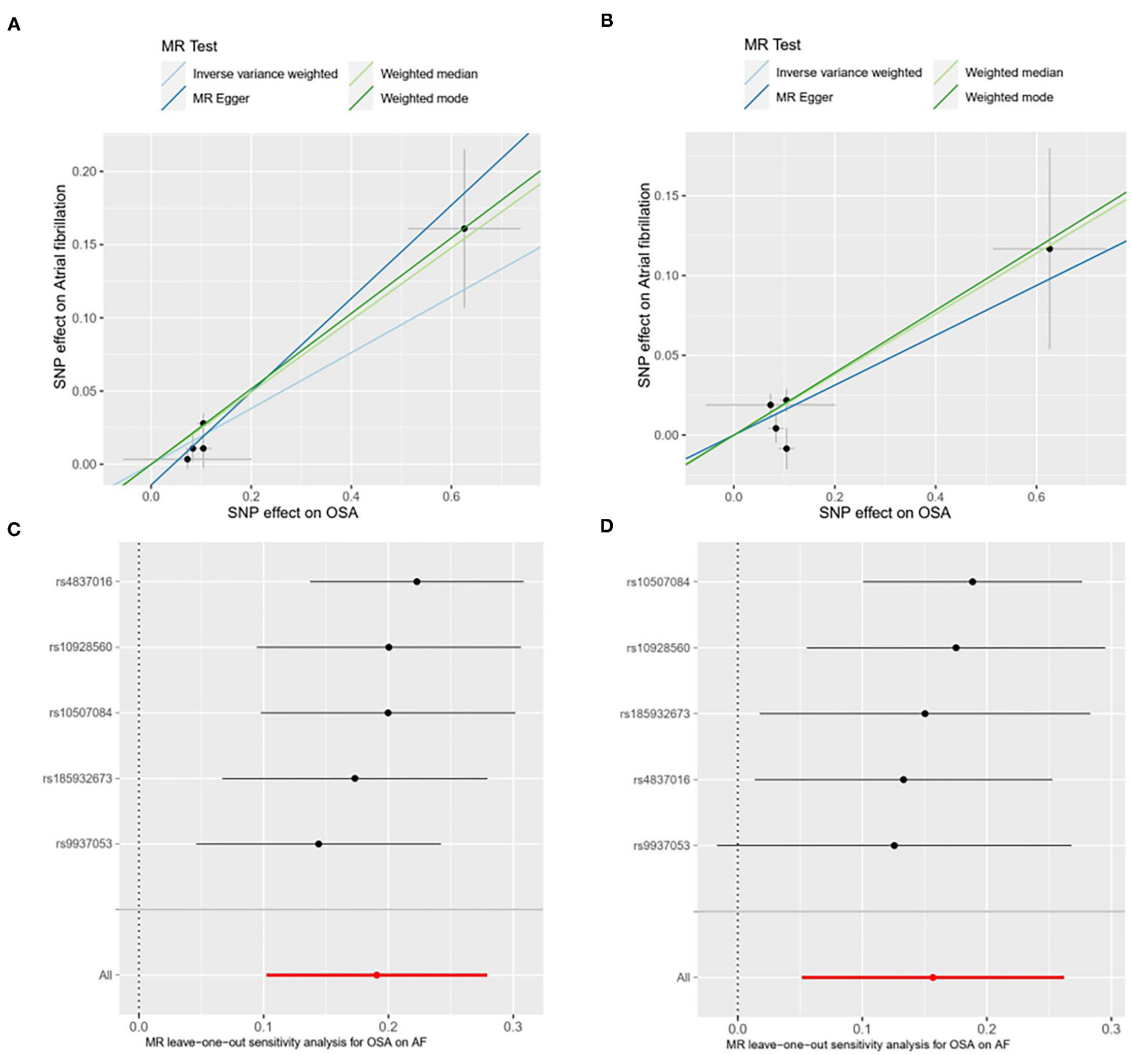
Scatter plot [primary analysis, **(A)**; replicated analysis, **(B)**] and leave-one-out test [primary analysis, **(C)**; replicated analysis, **(D)**] for genetically determined obstructive sleep apnea (OSA) on atrial firbrillation (AF) risk. SNP, single nucleotide polymorphisms; OSA, obstructive sleep apnea; AF, atrial fibrillation; MR, mendelian randomization.

We performed several sensitivity analyses aimed at estimating heterogeneity and horizontal pleiotropy in this MR analysis (Table 2). For the primary analysis, heterogeneity and horizontal pleiotropy were not observed in either Cochran's Q-test or MR Egger regression analysis (*P*_h_ = 0.386, *P*_intercept_ = 0.236). The results of the replicated analysis were consistent with the primary analysis, with no heterogeneity or horizontal pleiotropy found (*P*_h_ = 0.0895, P_intercept_ = 0.998). Besides, the MR-PRESSO results showed no outlier SNPs. In the leave-one-out analysis, we found that no single SNP was strongly driving the overall effect of OSA on AF in the primary analysis (Figure 2). But in the replicated analysis, we found that the SNP, rs9937053, will impact the overall results when it was removed.

#### Multivariable MR Analysis Results

**Figure 3:**
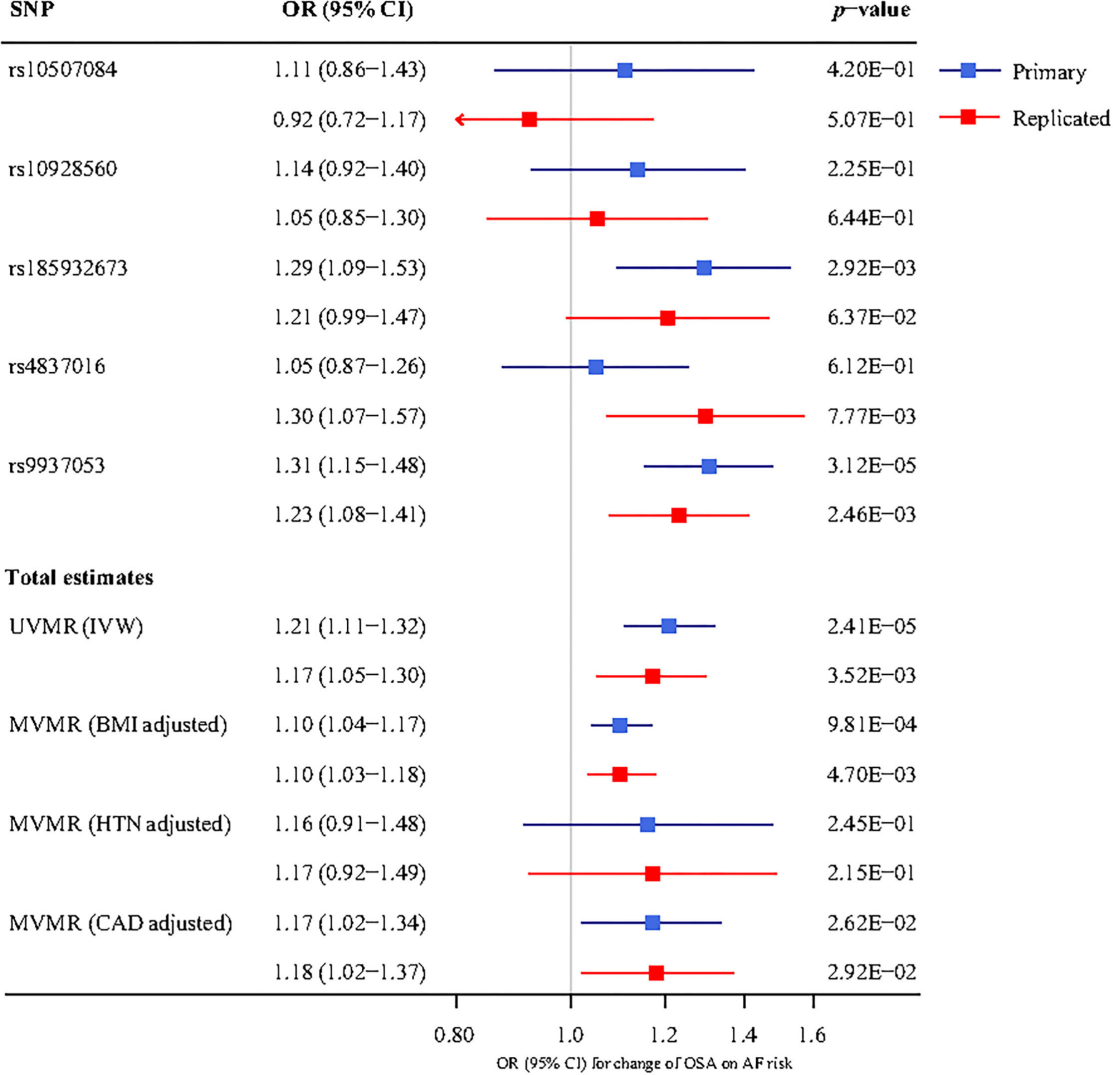
The association between genetically determined OSA and the AF risk using univariable and multivariable inverse-variance weighted (IVW) MR adjusted for body mass index (BMI), hypertension (HTN), and coronary artery disease (CAD). UVMR, univariable mendelian randomization; MVMR, multivariable mendelian randomization; SNP, single-nucleotide polymorphism; OR, odds ratio; CI, confidence interval; BMI, body mass index; HTN, hypertension; CAD, coronary artery disease; IVW, inverse-variance weighted; OSA, obstructive sleep apnea; AF, atrial fibrillation.

In the multivariable MR analysis, for the primary analysis, after adjusting for BMI, the relationship between OSA and risk of AF remains statistically significant (OR 1.10, 95%CI 1.04–1.17). And the adverse effect of OSA on AF existed after adjustment for CAD (OR 1.17, 95% CI 1.02–1.34). However, there was no evidence in favor of an association between OSA and AF after adjustment for hypertension (adjusted *P* = 0.245). The replicated analysis showed similar results to the primary analysis above (Figure 3).

## Discussion

With the MR method, we used genetic and clinical information, and provided the evidence to verify the causal association between the genetic determinants of OSA and the risk of AF, after adjusting for common cardiovascular factors. The major strength of our study is attributed to the large-scale sample, which supports a comprehensive analysis for incident AF and well-powered GWAS to obtain genetic instruments for MR analysis. Since AF remains the leading cardiac arrhythmia and one of the major causes of stroke, heart failure, and subsequent morbidity, our results provide further evidence from epidemiological significance advocating to emphasize the adequate control of OSA to reduce the global burden of AF and its complications.

In line with the current study, OSA was generally reported to be positively associated with the incidence of AF. Two novel pieces of research, using ECG monitoring in middle- or old-aged SA (Sleep Apnea) patients with obesity, found a 3 or 4.8% prevalence of nocturnal AF, higher than that of 0.4–1% in the general population (29, 30). As to longitudinal studies, Cadby (9) reported that the presence of OSA was associated with 1.55-fold of incident AF as measured by AF-related hospitalizations in a cohort of 6,841 middle-aged individuals with an average BMI of 30.7 kg/m2 during a median of 11.9 years of follow-up. Gami's (8) report found a two-fold higher risk of AF in patients with OSA in another large cohort with a similar average anthropometric index, however, a novel result showed that statistical difference lied only in the group of patients less than 65 years old. Conversely, in an age-stratified adjusted analysis, the OSA-AF association was not observed in 843 male cohorts with a mean age of 75 years (31). We speculated that epidemiological effects in the aforementioned studies modified were probably due to selection bias originating from limited sample size and study design. Moreover, despite epidemiological studies demonstrating the potential associations between OSA and AF, this evidence might be subject to residual confounding factors, as both OSA and AF shared several risk factors: at least 70% of OSA patients are obese (32), even MR analysis assessing the causality between genetic predicted BMI and OSA confirmed their strong genetic correlations (14), and a causal relationship was also identified between genetically predicted obesity and risk of AF (33, 34); so that obesity was an extremely important common factor as it was predisposed to result in the co-phenomenon of OSA and AF even in longitudinal studies. Apart from obesity, the habits of smoking (35, 36) and alcohol intake (37, 38) also tended to act as common risks of OSA and AF. With an instrumental variable analysis and a huge sample size, the MR approach can be used to study a cause-and-effect relationship without interferences of bias and confounding that are hard to circumvent in observational design, thus it is more appropriate for our exposure and outcome set. Chen (39) did an MR analysis to investigate the association between OSA and AF. They reported that OSA was related to an increased risk of AF. But they only did univariable analysis rather than multivariable analysis to adjust the potential confoundings such as BMI, CAD, and hypertension. Compared to their study, ours selected two outcome datasets for primary and secondary analysis. Therefore our results may be more robust.

There are several pathophysiologic mechanisms underlying the causal OSA-AF relationship. Apnea directly induces acidosis, hypoxia, hypercarbia, and elevated pulmonary pressure, subsequently resulting in combined sympathovagal activation contributing to hemodynamic and arrhythmogenic electrophysiologic changes, increasing the frequency of premature atrial contractions with the potential to initiate AF in a vulnerable substrate (40–42). Furthermore, long-term exposure to OSA leads to structural remodeling processes. It was observed in animals that intermittent airway obstruction produced connexin dysregulation, atrial fibrosis, atrial abnormal conduction, and increased AF susceptibility/duration (43). Accordingly, human atrial structural changes and conduction abnormalities occur during long-time OSA, forming a substrate for AF vulnerability (44, 45).

To note, some points in the data aroused our consideration. Effect sizes for the presence of OSA on the AF risk in our MR analysis were smaller compared with those obtained using observational data. This might be attributed to several reasons: part of AF is hard to capture when paroxysmal and might remain silent or asymptomatic in clinically stable patients or outpatients. Based on this background, cohort studies with closer follow-up of the participants had more opportunities to document more AF cases than those in our population. Meanwhile, another explanation is related to confounding-derived bias on the association between OSA and AF described above in observational studies. Multivariable MR association persisted after additional adjustment for comorbidities that may either mediate or confound the OSA-AF relationship, effect estimates were similar after BMI and CAD adjustment, but hypertension adjustment abolished the efficacy, suggesting that HTN might substantially mediate the effect of OSA on the risk of AF. It made sense that recognition already suggested that one of the OSA complications was a higher frequency of non-dipping blood pressure (BP) and pattern nocturnal hypertension (46), and genetic BP had a causal impact on AF (47).

Our study has some limitations. First, the limited number of instrumental SNPs in our study may distort MR estimates. But the F statistic of the SNP we used in this study were all >100, suggesting a strong correlation with OSA. Second, it is unlikely to remove all potential horizontal pleiotropy from the study, which may result in the biased estimation of causal inference. However, no pleiotropic effect was detected in the MR-Egger regression. Third, the specific ancestry of the samples also limits the generalizability of our results to other ancestries. Despite relative weaknesses, our study has several noteworthy advantages. First, we used MR method to estimate the causal effect between the OSA and AF, which contributes to the avoidance of measurement errors and residual confusion. In addition, we performed a series of sensitivity analyses to assess heterogeneity and pleiotropy, and we also conducted MVMR to correct for the effects of confounding factors including BMI, hypertension, and CAD.

## Conclusion

In summary, the current study provides the first causal evidence of the relationship between genetically predicted OSA and the risk of AF, suggesting that the therapy of OSA might represent an effective strategy to lower the incidence and re-occurrence of AF.

## Data Availability Statement

The original contributions presented in the study are included in the article/Supplementary Material, further inquiries can be directed to the corresponding author/s.

## Author Contributions

YLi, YLe, and RM drafted the manuscript. YLi and HT analyzed the data. PM, RM, and HT designed the study. PM and HY obtained the funding. All authors participated in the field survey and data collection, critically revised the manuscript, and gave final approval to the version submitted for publication.

## Funding

This work was supported by the National Natural Science Foundation of China (81974054), Liaoning Province's Xingliao talent program in China (No. XLYC1807116), and the Fundamental Research Funds for Central Universities of Central South University (2019zzts106).

## Conflict of Interest

The authors declare that the research was conducted in the absence of any commercial or financial relationships that could be construed as a potential conflict of interest.

## Publisher's Note

All claims expressed in this article are solely those of the authors and do not necessarily represent those of their affiliated organizations, or those of the publisher, the editors and the reviewers. Any product that may be evaluated in this article, or claim that may be made by its manufacturer, is not guaranteed or endorsed by the publisher.

## Supplementary Material

Supplementary Figure S1 Instrumental variable (IV) assumptions of MR.

Supplementary Table S1 Single-nucleotide polymorphisms associated with OSA.

Supplementary Table S2 Statistical power calculation for MR analyses.

## Abbreviations

OSA: obstructive sleep apnea
AF: atrial fibrillation
MR: mendelian randomization
GWAS: genome-wide association study
MVMR: multivariable mendelian randomization
BMI: body mass index
CAD: coronary artery disease
IVW: inverse-variance weighted
MR-PRESSO: MR-pleiotropy residual sum and outlier
OR: odds ratio
IV: instrumental variable
SNP: single-nucleotide polymorphism
LD: linkage disequilibrium
ECG: electrocardiogram
SA: sleep apnea
BP: blood pressure
HTN: hypertension.

## References

[1] Benjamin EJ Wolf PA D'Agostino RB Silbershatz H Kannel WB Levy D. Impact of atrial fibrillation on the risk of death: the Framingham Heart Study. Circulation. (1998) 98:946–52. 10.1161/01.CIR.98.10.946 9737513

[2] Chugh SS Roth GA Gillum RF Mensah GA. Global burden of atrial fibrillation in developed and developing nations. Glob Heart. (2014) 9:113–9. 10.1016/j.gheart.2014.01.004 25432121

[3] Goudis CA Ketikoglou DG. Obstructive sleep and atrial fibrillation: Pathophysiological mechanisms and therapeutic implications. Int J Cardiol. (2017) 230:293–300. 10.1016/j.ijcard.2016.12.120 28040290

[4] Kim YG Han KD Choi JI Choi YY Choi HY Boo KY. Non-genetic risk factors for atrial fibrillation are equally important in both young and old age: a nationwide population-based study. Eur J Prev Cardiol. (2020) 28:666–76. 10.1177/2047487320915664 34021574

[5] Linz D McEvoy RD Cowie MR Somers VK Nattel S Lévy P. Associations of Obstructive Sleep Apnea With Atrial Fibrillation and Continuous Positive Airway Pressure Treatment: A Review. JAMA Cardiol. (2018) 3:532–40. 10.1001/jamacardio.2018.0095 29541763

[6] Shamloo AS Dagres N Arya A Hindricks G. Atrial fibrillation: A review of modifiable risk factors and preventive strategies. Rom J Intern Med. (2019) 57:99–109. 10.2478/rjim-2018-0045 30648669

[7] Stevenson IH Teichtahl H Cunnington D Ciavarella S Gordon I Kalman JM. Prevalence of sleep disordered breathing in paroxysmal and persistent atrial fibrillation patients with normal left ventricular function. Eur Heart J. (2008) 29:1662–9. 10.1093/eurheartj/ehn214 18515807

[8] Gami AS Hodge DO Herges RM Olson EJ Nykodym J Kara T. Obstructive sleep apnea, obesity, and the risk of incident atrial fibrillation. J Am Coll Cardiol. (2007) 49:565–71. 10.1016/j.jacc.2006.08.060 17276180

[9] Cadby G McArdle N Briffa T Hillman DR Simpson L Knuiman M. Severity of OSA is an independent predictor of incident atrial fibrillation hospitalization in a large sleep-clinic cohort. Chest. (2015) 148:945–52. 10.1378/chest.15-0229 25927872

[10] Lin GM Colangelo LA Lloyd-Jones DM Redline S Yeboah J Heckbert SR. Association of sleep apnea and snoring with incident atrial fibrillation in the multi-ethnic study of atherosclerosis. Am J Epidemiol. (2015) 182:49–57. 10.1093/aje/kwv004 25977516 PMC4479113

[11] Huang B Liu H Scherlag BJ Sun L Xing S Xu J. Atrial fibrillation in obstructive sleep apnea: Neural mechanisms and emerging therapies. Trends Cardiovasc Med. (2021) 31:127–32. 10.1016/j.tcm.2020.01.006 32008837

[12] Li X Zhou X Xu X Dai J Chen C Ma L. Effects of continuous positive airway pressure treatment in obstructive sleep apnea patients with atrial fibrillation: a meta-analysis. Medicine (Baltimore). (2021) 100:e25438. 10.1097/MD.0000000000025438 33847645 PMC8051983

[13] Burgess S Thompson SG. Multivariable Mendelian randomization: the use of pleiotropic genetic variants to estimate causal effects. Am J Epidemiol. (2015) 181:251–60. 10.1093/aje/kwu283 25632051 PMC4325677

[14] Smith GD Ebrahim S. 'Mendelian randomization': can genetic epidemiology contribute to understanding environmental determinants of disease? Int J Epidemiol. (2003) 32:1–22. 10.1093/ije/dyg070 12689998

[15] Strausz S Ruotsalainen S Ollila HM Karjalainen J Kiiskinen T Reeve M. Genetic analysis of obstructive sleep apnoea discovers a strong association with cardiometabolic health. Eur Respir J. (2021) 57. 10.1101/2020.08.04.235994 33243845

[16] Burgess S Thompson SG. Avoiding bias from weak instruments in Mendelian randomization studies. Int J Epidemiol. (2011) 40:755–64. 10.1093/ije/dyr036 21414999

[17] Yengo L Sidorenko J Kemper KE Zheng Z Wood AR Weedon MN. Meta-analysis of genome-wide association studies for height and body mass index in ~700000 individuals of European ancestry. Hum Mol Genet. (2018) 27:3641–9. 10.1093/hmg/ddy271 30124842 PMC6488973

[18] Nikpay M Goel A Won HH Hall LM Willenborg C Kanoni S. A comprehensive 1,000 Genomes-based genome-wide association meta-analysis of coronary artery disease. Nat Genet. (2015) 47:1121–30. 10.1038/ng.3396 26343387 PMC4589895

[19] Nielsen JB Thorolfsdottir RB Fritsche LG Zhou W Skov MW Graham SE. Biobank-driven genomic discovery yields new insight into atrial fibrillation biology. Nat Genet. (2018) 50:1234–9. 10.1038/s41588-018-0171-3 30061737 PMC6530775

[20] Roselli C Chaffin MD Weng LC Aeschbacher S Ahlberg G Albert CM. Multi-ethnic genome-wide association study for atrial fibrillation. Nat Genet. (2018) 50:1225–33. 10.1038/s41588-018-0133-9 29892015 PMC6136836

[21] Burgess S Bowden J Fall T Ingelsson E Thompson SG. Sensitivity analyses for robust causal inference from mendelian randomization analyses with multiple genetic variants. Epidemiology. (2017) 28:30–42. 10.1097/EDE.0000000000000559 27749700 PMC5133381

[22] Bowden J Davey Smith G Haycock PC Burgess S. Consistent estimation in mendelian randomization with some invalid instruments using a weighted median estimator. Genet Epidemiol. (2016) 40:304–14. 10.1002/gepi.21965 27061298 PMC4849733

[23] Bowden J Davey Smith G Burgess S. Mendelian randomization with invalid instruments: effect estimation and bias detection through Egger regression. Int J Epidemiol. (2015) 44:512–25. 10.1093/ije/dyv080 26050253 PMC4469799

[24] Greco MF Minelli C Sheehan NA Thompson JR. Detecting pleiotropy in Mendelian randomisation studies with summary data and a continuous outcome. Stat Med. (2015) 34:2926–40. 10.1002/sim.6522 25950993

[25] Tedrow UB Conen D Ridker PM Cook NR Koplan BA Manson JE. The long- and short-term impact of elevated body mass index on the risk of new atrial fibrillation the WHS (women's health study). J Am Coll Cardiol. (2010) 55:2319–27. 10.1016/j.jacc.2010.02.029 20488302 PMC2880879

[26] Aronow WS. Hypertension associated with atrial fibrillation. Ann Transl Med. (2017) 5:457. 10.21037/atm.2017.10.33 29285490 PMC5733333

[27] Goto S Bhatt DL Röther J Alberts M Hill MD Ikeda Y. Prevalence, clinical profile, and cardiovascular outcomes of atrial fibrillation patients with atherothrombosis. Am Heart J. (2008) 156:855–63. 10.1016/j.ahj.2008.06.029 19061698

[28] Verbanck M Chen CY Neale B Do R. Detection of widespread horizontal pleiotropy in causal relationships inferred from Mendelian randomization between complex traits and diseases. Nat Genet. (2018) 50:693–8. 10.1038/s41588-018-0099-7 29686387 PMC6083837

[29] Guilleminault C Connolly SJ Winkle RA. Cardiac arrhythmia and conduction disturbances during sleep in 400 patients with sleep apnea syndrome. Am J Cardiol. (1983) 52:490–4. 10.1016/0002-9149(83)90013-9 6193700

[30] Mehra R Benjamin EJ Shahar E Gottlieb DJ Nawabit R Kirchner HL. Association of nocturnal arrhythmias with sleep-disordered breathing: the sleep heart health study. Am J Respir Crit Care Med. (2006) 173:910–6. 10.1164/rccm.200509-1442OC 16424443 PMC2662909

[31] May AM Blackwell T Stone PH Stone KL Cawthon PM Sauer WH. Central sleep-disordered breathing predicts incident atrial fibrillation in older men. Am J Respir Crit Care Med. (2016) 193:783–91. 10.1164/rccm.201508-1523OC 26595380 PMC4824932

[32] Tuomilehto H Seppä J Uusitupa M. Obesity and obstructive sleep apnea–clinical significance of weight loss. Sleep Med Rev. (2013) 17:321–9. 10.1016/j.smrv.2012.08.002 23079209

[33] Chatterjee NA Giulianini F Geelhoed B Lunetta KL Misialek JR Niemeijer MN. Genetic obesity and the risk of atrial fibrillation: causal estimates from mendelian randomization. Circulation. (2017) 135:741–54. 10.1161/CIRCULATIONAHA.116.024921 27974350 PMC5322057

[34] Larsson SC Bäck M Rees JMB Mason AM Burgess S. Body mass index and body composition in relation to 14 cardiovascular conditions in UK Biobank: a Mendelian randomization study. Eur Heart J. (2020) 41:221–6. 10.1093/eurheartj/ehz388 31195408 PMC6945523

[35] Lin YN Li QY Zhang XJ. Interaction between smoking and obstructive sleep apnea: not just participants. Chin Med J. (2012) 125:3150–6. 10.3760/cma.j.issn.0366-6999.2012.17.033 22932197

[36] Larsson SC Mason AM Bäck M Klarin D Damrauer SM Michaëlsson K. Genetic predisposition to smoking in relation to 14 cardiovascular diseases. Eur Heart J. (2020) 41:3304–10. 10.1093/eurheartj/ehaa193 32300774 PMC7544540

[37] Taveira KVM Kuntze MM Berretta F de Souza BDM Godolfim LR Demathe T. Association between obstructive sleep apnea and alcohol, caffeine and tobacco: a meta-analysis. J Oral Rehabil. (2018) 45:890–902. 10.1111/joor.12686 29971810

[38] Lu Y Guo Y Lin H Wang Z Zheng L. Genetically determined tobacco and alcohol use and risk of atrial fibrillation. BMC Med Genomics. (2021) 14:73. 10.1186/s12920-021-00915-0 33750369 PMC7944892

[39] Chen W Cai X Yan H Pan Y. Causal effect of obstructive sleep apnea on atrial fibrillation: a mendelian randomization study. J Am Heart Assoc. (2021) 10:e022560. 10.1161/JAHA.121.022560 34796736 PMC9075405

[40] Linz D Hohl M Ukena C Mahfoud F Wirth K Neuberger HR. Obstructive respiratory events and premature atrial contractions after cardioversion. Eur Respir J. (2015) 45:1332–40. 10.1183/09031936.00175714 25745047

[41] Ghias M Scherlag BJ Lu Z Niu G Moers A Jackman WM. The role of ganglionated plexi in apnea-related atrial fibrillation. J Am Coll Cardiol. (2009) 54:2075–83. 10.1016/j.jacc.2009.09.014 19926016

[42] Yu L Li X Huang B Zhou X Wang M Zhou L. Atrial fibrillation in acute obstructive sleep apnea: autonomic nervous mechanism and modulation. J Am Heart Assoc. (2017) 6. 10.1161/JAHA.117.006264 28903938 PMC5634281

[43] Iwasaki YK Kato T Xiong F Shi YF Naud P Maguy A. Atrial fibrillation promotion with long-term repetitive obstructive sleep apnea in a rat model. J Am Coll Cardiol. (2014) 64:2013–23. 10.1016/j.jacc.2014.05.077 25440097

[44] Dimitri H Ng M Brooks AG Kuklik P Stiles MK Lau DH. Atrial remodeling in obstructive sleep apnea: implications for atrial fibrillation. Heart Rhythm. (2012) 9:321–7. 10.1016/j.hrthm.2011.10.017 22016075

[45] Anter E Di Biase L Contreras-Valdes FM Gianni C Mohanty S Tschabrunn CM. Atrial substrate and triggers of paroxysmal atrial fibrillation in patients with obstructive sleep apnea. Circ Arrhythm Electrophysiol. (2017) 10. 10.1161/CIRCEP.117.005407 29133380 PMC5726812

[46] Pio-Abreu A Moreno HJr Drager LF. Obstructive sleep apnea and ambulatory blood pressure monitoring: current evidence and research gaps. J Hum Hypertens. (2021) 35:315–24. 10.1038/s41371-020-00470-8 33414503

[47] Georgiopoulos G Ntritsos G Stamatelopoulos K Tsioufis C Aimo A Masi S. The relationship between blood pressure and risk of atrial fibrillation: a Mendelian randomization study. Eur J Prev Cardiol. (2021) 28:1617. 10.1093/eurjpc/zwab005 33556963

