# Supplementary material for: Assessment of the Causal Effects of Obstructive Sleep Apnea on Atrial Fibrillation: A Mendelian Randomization Study
Source: Front Cardiovasc Med. 2022 Feb 11;9:843681. doi: 10.3389/fcvm.2022.843681 (PMC8874127; doi:10.3389/fcvm.2022.843681)
Supplement: Supplementary Figure S1 — Instrumental variable (IV) assumptions of MR. [file Data_Sheet_1.docx]

**Supplementary**

Figure S1. Instrumental variable (IV) assumptions of Mendelian randomization.

Table S1. Single-nucleotide polymorphisms associated with obstructive sleep apnea.

Table S2. Statistical power calculation for Mendelian randomization analyses.

**
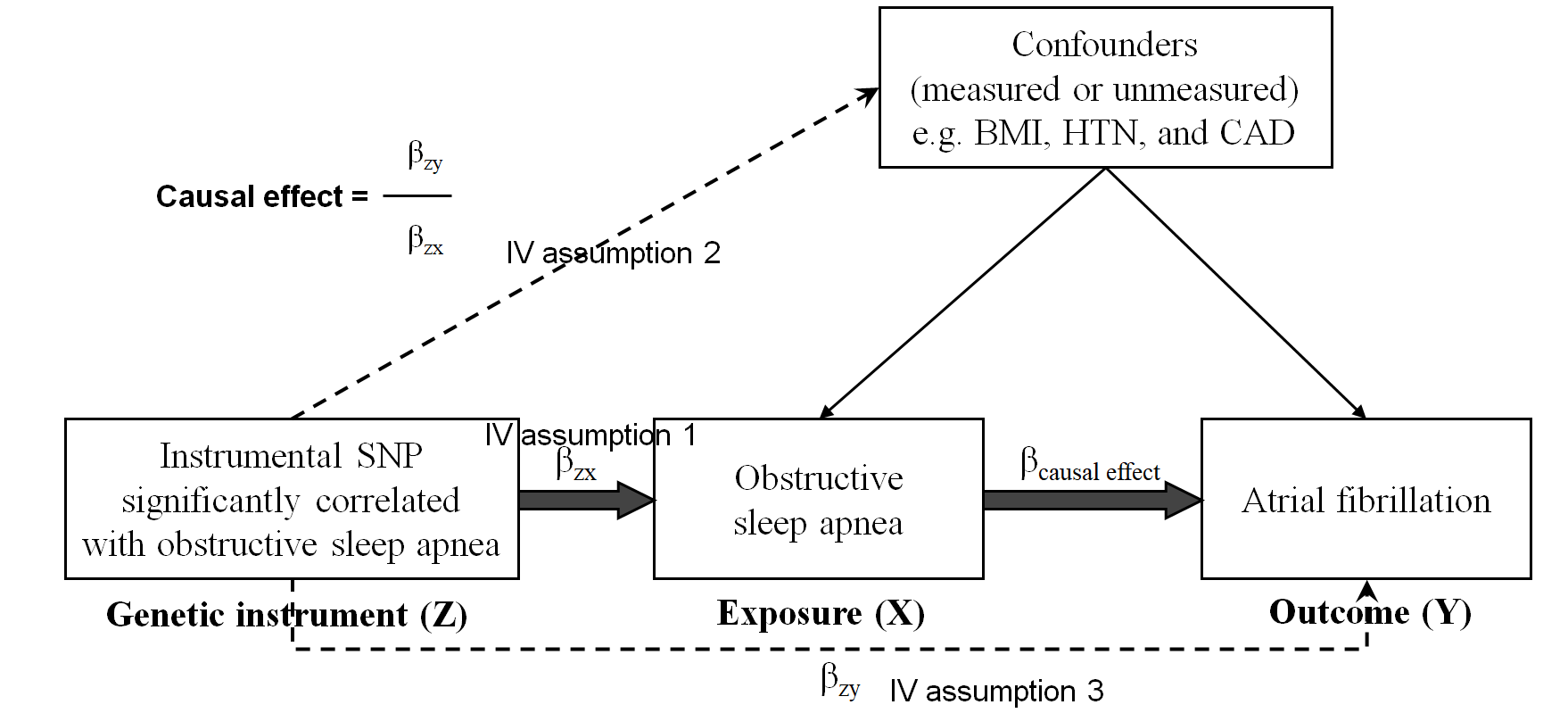
**

**Figure S1. Instrumental variable (IV) assumptions of Mendelian randomization.**

SNP: Single nucleotide polymorphism; BMI:body mass index; HTN: hypertension; CAD: coronary artery disease; IV: Instrumental variable

**Table S1. Single-nucleotide polymorphisms associated with obstructive sleep apnea.**

|  |  |  |  |  |  |  |  | **OSA** |  |  |  | **AF** |  |
| --- | --- | --- | --- | --- | --- | --- | --- | --- | --- | --- | --- | --- | --- |
| **CHR** | **Position** | **SNP** | **OA** | **EA** | **Genes** | ***F-*stat** | **Beta** | **SE** | ***p*-value** |  | **Beta** | **SE** | ***p*-value** |
| 16 | 53765595 | rs9937053 | G | A | *FTO* | 772 | 0.10436 | 0.0128 | 4.30E-16 |  | 0.0279 | 0.0067 | 3.05E-05 |
| 12 | 97359374 | rs10507084 | C | T | *RMST/NEDD1* | 630 | 0.10436 | 0.0157 | 2.80E-11 |  | 0.0108 | 0.0134 | 4.19E-01 |
| 10 | 12656440 | rs185932673 | C | T | *CAMK1D* | 527 | 0.625938 | 0.1122 | 2.40E-08 |  | 0.161 | 0.0541 | 2.92E-03 |
| 9 | 125379530 | rs4837016 | G | A | *GAPVD1* | 535 | -0.07257 | 0.1282 | 1.50E-08 |  | -0.0034 | 0.0067 | 6.12E-01 |
| 2 | 136234237 | rs10928560 | C | T | *CXCR4* | 525 | -0.08338 | 0.01501 | 2.80E-08 |  | -0.0108 | 0.0089 | 2.27E-01 |

#### SNP: Single nucleotide polymorphisms; OSA:obstructive sleep apnea; AF: [atrial fibrillation](C:/Users/LYL/AppData/Local/youdao/dict/Application/8.9.9.0/resultui/html/index.html#/javascript:;);

**Table S2. Statistical power calculation for Mendelian randomization analyses.**

|  |  |  |  | **Statistical power at the given odds ratio** | | | | | | |  |
| --- | --- | --- | --- | --- | --- | --- | --- | --- | --- | --- | --- |
| **Exposure** | **Outcome** | **Sample size** | **Cases** | **OR = 0.80** | **OR = 0.90** | **OR = 0.95** | **OR = 1.05** | **OR = 1.10** | **OR = 1.20** | **OR = 1.30** | **OR = 1.50** |
| OSA-5 SNPs | AF | 1,030,836 | 60,620 | 1.00 | 1.00 | 0.93 | 0.93 | 1.00 | 1.00 | 1.00 | 1.00 |
| OSA-5 SNPs | AF | 537,409 | 55,114 | 1.00 | 1.00 | 0.90 | 0.89 | 1.00 | 1.00 | 1.00 | 1.00 |

OSA:obstructive sleep apnea; AF: [atrial fibrillation](C:/Users/LYL/AppData/Local/youdao/dict/Application/8.9.9.0/resultui/html/index.html#/javascript:;); SNP: Single nucleotide polymorphism; OR: odds ratio
